# Seeking Health Information and Social Support in the Diabetes Online Community

**DOI:** 10.3389/fcdhc.2021.708405

**Published:** 2021-09-28

**Authors:** Allyson Hughes, Nazanin Heydarian, Diana Gerardo, Isabela Solis, Osvaldo Morera

**Affiliations:** ^1^ Department of Primary Care, Ohio University Heritage College of Osteopathic Medicine, Athens, OH, United States; ^2^ Department of Social Work, School of Social Work, University of Texas Rio Grande Valley, Edinburg, TX, United States; ^3^ Department of Psychology, The University of Texas at El Paso, El Paso, TX, United States

**Keywords:** type 1 diabetes, social support, seeking health information, scale development, continuous glucose monitoring, insulin pump

## Abstract

**Purpose:**

People with type 1 diabetes (T1D) search for health information online in the Diabetes Online Community (DOC), where individuals with diabetes, researchers and caregivers post and respond to health questions. The aims of this study were 1) to understand how people with T1D are seeking health information and engaging in health behaviors in the DOC, and 2) develop a measure of online health information seeking in adults with T1D.

**Research Method:**

Ninety-five adults with T1D completed qualitative prompts online.

**Results:**

Themes that emerged in this study included sense of community, and multiple types of social support that are necessary in disease management.

**Conclusions:**

This study used qualitative methods to develop a valid scale tailored for adults with T1D. Future research should seek to collect additional data to bolster validity and reliability.

## Introduction

Seeking health information and engagement in online health communities are significant and emerging public health phenomena. Health information is being exchanged continuously in the Diabetes Online Community (DOC; 1). The online community thrives on social media sites and provides largely anecdotal evidence (microblogging *via* tweets, Facebook posts, blog posts and discussion boards) regarding medical decision-making (2). Type 1 Diabetes (T1D) is a chronic condition that requires checking blood glucose levels multiple times a day, multiple insulin injections daily, and/or use of durable medical equipment that provides the person with diabetes with insulin (3). T1D management varies from one person to the next; health practices that work extremely well for one person may be ineffective for another person (4). People with T1D, who are members of the DOC, can encounter health information (considered one directional), but also participate in health engagement (bidirectional information exchanges) (5).

The need for tailored health information often leads people with T1D to seek information from their peers in a phenomenon known as peer-to-peer healthcare (6). Health information seeking has long been documented as a key coping strategy and is characterized as a monitoring behavior that leads to problem-focused coping strategies (7). Findings show increased information seeking is associated with increased self-care and health promotion (8). Additionally, sharing health information is positively associated with behavioral intentions to follow health recommendations (9). It is well established that the DOC is a environment for peer support disease management support (10). Although we know about peer health information seeking and its benefits, and the way the DOC is set up would lend itself nicely to peer information seeking, no one has yet examined peer health information seeking in the DOC before.

Benefits from being a member of the DOC include 1) increased positive emotional experiences, 2) increased positive attitudes toward T1D, and 3) increased engagement in T1D management behaviors (11). Psychosocial benefits to participating in online support groups include empowerment and social support (12). Indeed, in a previous study, diabetes bloggers perceived more social support the more they blogged (13). In a recent qualitative study, participants with either T1D or type 2 diabetes described the benefits of DOC membership and one key benefit was the ability for the online community to feel like a tight knit family (14). The diabetes online community can also promote facilitators of diabetes self-management such as positive individual strategies and social support from peers and healthcare providers (15). The online community could be the key for providing diabetes self-management strategies outside of the clinic (16).

Although there is much existing research on psychosocial and behavioral benefits of the DOC, there is a dearth of information on health information seeking in the DOC. For this reason, the current qualitative study was exploratory in nature and sought to determine how people with T1D seek health information and participate in health engagement in the DOC and create a scale on seeking health information online for adults with T1D.

## Methods

This study’s qualitative data was collected solely online using Qualtrics and was analyzed using a thorough thematic analysis framework (17 **).**

### Participants

Participants were recruited from the DOC *via* Facebook posts, tweets using the hashtags #doc, #type1 diabetes and #dsma, and peer to peer referrals. Participant eligibility required the following: 1) being 18 years of age or older, 2) being a member of the DOC and 3) having been diagnosed with T1D by a doctor. The study sample included 95 DOC stakeholders. Prior to data collection, the study underwent review by the Institutional Review Board at the University of Texas at El Paso.

### Measures

The demographics questionnaire asked participants to self-report their age, gender, marital status, ethnicity, and education level. Participants reported their diabetes duration, mode of insulin delivery and health outcomes. Participants were provided with qualitative questions in order to better understand their role in the DOC and their experience in the DOC in general. Participants answered 15 Likert scale questions from the Attitudes towards Seeking Health Information Online Scale. Sample items included: I frequently use the internet to gain health advice in the Diabetes Online Community; I review multiple internet sources in the Diabetes Online Community before making a health decision for myself; I do not follow the health information that I find on social media in the Diabetes Online Community; I trust the health information that I find in the Diabetes Online Community; I feel comfortable receiving health advice in the Diabetes Online Community; I trust the health information that my friends on social media (Facebook, Twitter, Instagram, discussion forums) provide in the Diabetes Online Community. The scale was shown to the participants and they provided qualitative feedback regarding item wording. Existing literature was used to develop the scale and the qualitative questions further refined the scale with an emphasis on improving validity.

### Qualitative Research Questions

Participants responded to open-ended questions about their experiences as members of the DOC. The goal of these questions was to determine: *What are people with T1D’s perceptions of how the DOC assists them with their physical and mental health*? The second question was:** **W*hat characterizes a person with T1D’s experiences interacting with DOC members to make a treatment decision?* The third question was: *What elements of the DOC do people with T1D find to be most useful?* These research questions were formulated in relation to the open-ended questions participants answered.

### Data Analysis

#### Research Assistant Training

In order to promote validity and reliability of the findings, two research assistants (RA) were trained to code the qualitative data. The RA met with the principal investigator several times to practice coding. The research team discussed the benefits and limitations of mixed methods in the online setting and discussed assigned readings, YouTube videos and podcasts regarding the culture of T1D in adults.

#### Thematic Analyses Plan

A codebook was developed based upon existing literature and exploratory themes derived from RA training materials. The codebook consisted of 1) proposed categories, 2) proposed themes, 3) proposed subthemes, 4) definitions, and 5) example quotes to illustrate the meaning of the themes. Categories were populated by themes and themes were populated by subthemes. Themes and subthemes were made up of codes and these were derived from participant quotes. There were two coders, authors ASH and DG. After each round of coding, the authors would compare their codes and discuss discrepancies and come to agreement. Themes were determined both inductively and deductively using not only existing literature but also the data to structure themes.

## Results

### Participant Characteristics

Ninety-five participants were included in this sample. Complete demographics are available in **Table 1**.

**Table 1:** Demographics.

Age, Mean (SD)	26.8 (7.18)
Gender, N (%)	
Female	87 (91.6%)
Race, N (%)	
White	75 (78.9%)
African-American	2 (2.1%)
Hispanic or Latino	12 (12.6%)
Other	6 (6.3%)
Education, N (%)	
< Bachelor’s Degree	34 (35.8%)
Bachelor’s Degree	34 (35.8%)
> Bachelor’s Degree	27 (28.4%)
Household Income, Mean (SD)	$66,283 (56,148)
*Diabetes Demographics*	
Insulin Pump, N (%)	59 (62.1%)
Length of Diagnosis, Mean (SD)	12.3 years (9.17)
Continuous Glucose Monitor, N (%)	55 (57.9%)
A1c, Mean (SD)	7.1% (1.5)

### Thematic Analyses

Coding categories included 756 quotes, and 36 themes. Participants completed a detailed assessment of the Attitudes Toward Seeking Online Health Information scale. More than half of the participants (56.6%) reported completing the scale in 15 minutes or less. Seventy-seven percent of the participants believed the questions were written by someone who had an accurate idea of T1D. The first theme was *sense of community* (*n* = 48), where participants reported experiencing an overall feeling of belonging to something “bigger” in the DOC. One example of this is: [ID 177]: “Reading other people’s stories whom I can relate with. No judgement and everyone understands each other”. The second theme was *social interaction and support* (*n* = 30), defined as DOC members engaging with other members and receiving social support during these interactions. The third theme was *informational support* (*n* = 29), participants reported exchanging advice, and receiving and sharing suggestions related to diabetes management and overall information. Participants also reported learning about new technologies and medications.

### Pros and Cons of Membership in the DOC

Participants were also asked to describe the pros of being a member of the DOC. Themes included gaining *information and advice from other members of the DOC* (*n* = 25), experiencing a “*sense of community*” within the DOC (*n* = 52), stating [ID 108]: “We are all going through this together, so that is the best part”. Participants also described cons of being a member of the DOC including *comparing self to others (n* = 10) and *misinformation* (*n* = 6).

### Physical Impact of the DOC

Overall participants stated that the DOC had a *positive impact on their physical health* (*n* = 64), stating improvements in self-care, exercise behaviors, improved nutrition, and access to healthcare and medication. Quotes included: [ID 109] “It’s improved. I’ve learned a few tidbits to apply to daily life, especially about alternative snacking habits and insulin dosing strategies”. Another participant stated that the DOC helped them adjust to a new lifestyle change: [ID 120]: “Being able to see what others do in regards to their diet and preventing highs and lows before, during and after workouts has been amazing”. Lastly, a participant described a dire experience [ID 155]: “At one point I had no insulin and as soon as I asked for help someone from the group quickly got in contact with me and sent me some right away”.

### Mental Health Impact of the DOC

The majority of participants endorsed that the DOC had a *positive impact on their mental health* including feeling less isolated. Mental health benefits included receiving encouragement, feeling less alone, having an improved mental health status, feeling a sense of community, and normalizing the diabetes experience. Of note, participants also stated the *negative mental health impact* that the DOC can impart such that being part of the DOC caused them to experience anxiety about T1D and that involvement in the DOC promoted negative behaviors. An example of this negative behavior occurs when a member of the DOC shares a picture of what their blood sugars have been in the last 24 hours. If they post a picture of blood sugars in target range then that may produce anxiety in DOC members who are not experiencing in range blood sugars.

### Seeking Health Information in the DOC

Several participants reported *seeking out advice about dosing insulin* (*n* = 11). Overall, participants described seeking advice about *durable medical equipment* (*n* = 16). There were few overlapping themes regarding topics participants sought advice and engagement about: *insulin dosing while exercis*ing (*n* = 9), *allergic reactions* (*n* = 1), *blood glucose advice* (*n* = 3), *diabulimia treatment* (*n* = 1), *blood sugar meter advice* (*n* = 1), *nutrition* (*n* = 2), and *sick days* (*n* = 1). Others described consulting not only their healthcare professional but also the online community [*doctor and DOC* (*n* = 1)] and *giving advice* (*n* = 5). A participant described seeking help from the DOC when they were in the middle of a medical emergency in a foreign country.

Participants also reported seeking existing advice in order to answer any health questions they may have. Members of the DOC may endorse the answer to the question or state how this piece of information has impacted them. Participants reported that the DOC provided them with information on how to use their medical devices, including managing sick days, emergency situations, and exercising. Some DOC members indicated that they provide advice to others but they do not request it. This dynamic has important implications for the way that individuals with chronic disease seek health information. Specifically, existing information seeking theories do not take into account the role of information brokering that occurs in the DOC. Furthermore, there is a difference between the types of information (by topic) that are being sought and how (actively seeking advice *versus* passively seeking advice) they are being sought.

### Stakeholder Assessment of Seeking Health Information Online Scale

Regarding additional comments about the questionnaire, participants reported support for the items and endorsed the cultural competency of the scale with statements such as: [ID 195]: “asked relevant questions for someone with T1D”, and [ID 149]: “Asking how the diabetes community has helped with physical and mental health. Those are 2 significant aspects that are affected by this condition”. Several participants endorsed Instagram as part of the DOC because they sought social support for T1D management on Instagram. Instagram had not been previously included in the survey materials.

Specific participant requests encompassed a need to improve clarity and change “treatment decisions” to “advice”. Many participants stated the importance and major impact of the DOC in how they make decisions about which medical devices they will be using. Much of the conversation in the DOC involves medical device usage, tips and tricks, navigating insurance and medical claims advice and overall conversations on accessibility. Breaking news about medical devices is often shared widely in the DOC such that when FDA approval is given to a new diabetes device, DOC members will find out from social media-based news outlets and other DOC members before they find out from their doctor. Of importance, participants stated that they began using specific types of durable medical equipment due to endorsements from DOC members.

Participants also reported seeking existing advice in order to answer any health questions they may have. Members of the DOC may endorse the answer to the question or state how this piece of information has impacted them. This dynamic challenges how online health information seeking was originally conceptualized for this set of studies. The initial conceptualization did not account for existing information but instead focused on sharing new information.

Participants requested more open-ended survey questions and more studies about various aspects of information seeking in the DOC. Participants in this community are very forthcoming in what they need and want to see in research. Overall, participants stated that the questions seemed relevant to T1D and DOC usage. They reported gathering information first in preparation for making a decision about whether or not to go to the doctor. Participants reported that the DOC provided them with information on how to use their medical devices, including information about how to address treatment management for sick days, emergencies situations, and exercising.

An interesting phenomenon within these data (and generalized to this community) is that some DOC members indicated that they provide advice to others but they do not request it. Key quotes included: [ID 189]: “I wouldn’t make a treatment decision online with someone who I do not know as that could result in poor treatment. I have made suggestions once in a while or advised how I would treat myself in that situation”. and [ID 173]: “A woman had asked about using her libre [continuous glucose monitor] to make decisions on the insulin, she was new to it and was hesitant about how to treat. I gave her several personal examples and showed successes and failures. Others did the same thing. She decided to try small changes and let her doctor know which I also advised.” Another key quote: [ID 108]:

I have a family in CO who has a son about the same age as my son who was having a hard time reach out to me direct, and I was able to help them get some things set while their son had the flu. It was a really good feeling and we have been friends for some time now. As for me, I haven’t had to ask for help on anything in a long time since I have done most of it on my own for so long.

This dynamic has important implications for the way that individuals with chronic disease seek health information. Specifically, existing information seeking theories do not take into account the role of information brokering that occurs in the online community. Furthermore, there is a difference between the types of information (by topic) that are being sought and how actively seeking advice *versus* passively seeking advice) they are being sought.

Participants had a wide range of experience and weekly commitment giving medical advice in the DOC. An example of this range was that some participants stated that they spent 0 minutes weekly providing advice in the DOC but others stated that they spent 6 to 7 hours giving advice in the DOC per week. Of note, one participant indicated: [ID 103] “Not much. While I appreciate anecdotal advice but I prefer medical information to come from my endocrinologist. I rarely SEEK out medical advice … that doesn’t prevent it from being offered to me though….” Several participants stated that they were recipients of unsolicited health advice in the DOC. Another identified: [ID 109]: “whatever time I don’t spend looking, I am helping”. Another participant stated, [ID 179]: “Currently, I am not seeking advice, at least not 100% of the time. Sometimes I’m just reading and stumble upon advice that I find useful”.

## Discussion

The study examined seeking health information online and behavior engagement in the DOC, a prominent diabetes focused health community where peers provide multiple types of social support and broker information. The DOC assists with a variety of issues (including information gathering, with medical devices, promoting social support and connecting others). Social support sought after in the DOC included emotional support, encouragement to get a continuous glucose monitor, informational support and inspiration. Existing DOC literature does not examine the phenomenon of health information seeking so this research was critical for developing and further validating the scale.

The majority of participants expressed that the DOC had a positive impact on their physical health, stating improvements in self-care, exercise behaviors, nutrition, and access to healthcare and medication. Participants also reported receiving encouragement, feeling less alone, having an improved mental health status, feeling a sense of community, and normalizing the diabetes experience. Participants endorsed that the DOC had a positive impact on their mental health. Importantly, a handful of participants reported experiencing anxiety related to the DOC which appears to be connected to the behavior of comparing oneself to other members of the DOC.

Many participants engaged in health behavior resources. Participants sought advice about medication dosing and using insulin pumps and continuous glucose monitors. There were very few overlapping topics which garnered further support for the complexity of the needs in the DOC and the difficulty of disease management. Several participants reported not seeking information online but instead providing information online. These diverse responses show that seeking health information in the DOC is not for everyone but those who do seek the health information benefit greatly. The DOC is capable of providing important, tailored information and assistance.

Participants were very expressive in what was the most useful part of the DOC such as informational support where DOC members are exchanging advice about disease management. Participants also expressed the importance of social interaction and support where DOC members are interacting with other members and receiving social support during these interactions. The final theme was the sense of community experienced by members of the DOC. Participants expressed feeling part of a larger group where they do not feel judged and they related to other members while reaping benefits of said membership. Overall, these examples and themes provide powerful support that the DOC has a beneficial impact on the amount of social support that individuals with T1D are experiencing. At the core of this research, is the need to further understand how individuals with T1D are gaining health information in the DOC and the impact that this support has on their health.

Regarding how information seeking is occurring in the DOC, participants also reported following advice that already existed in social media such that they are not generating a new post to find an answer to their question. Instead, they are seeking existing posts where their health question has been answered. Most social media sites have a search mechanism that makes this fast and easy to accomplish. Importantly, members of the DOC also reported on the phenomenon of endorsing existing answers which impacts the trustworthiness of the information. This dynamic greatly challenges how online health information seeking is presently studied in the literature (using the existing scales with vignettes based on hypothetical situations). Of importance, participants stated that they began using specific types of durable medical equipment because of endorsements from DOC members.

This project provides a view of the “real world” perspective T1D management outside of the health clinic. The project also sought to clarify how members of the DOC seek health information and what they perceive to be the benefits of being a member. Prior research has suggested evidence of benefits of membership include emotional support and informational support (18). Previous research has also suggested anecdotal evidence of benefits of membership include increased positive emotional experiences, increased positive attitudes towards T1D, and increased engagement in T1D management behaviors (1).

These findings provide support for the four key types of social support: *emotional support* (e.g., providing caring endearments when needed), *informational support* (e.g., providing advice about how much insulin to dose during exercise), *instrumental support* (e.g., providing insulin pump training to individuals who do not have the local training resources), and *appraisal support* (e.g., members make other feel “normal”). Findings also provided support for social provisions: *guidance* (e.g., advice about treatment decisions), *reliable alliance* (e.g., guarantees that others will be there in a stressful situation such as being without insulin or when an insulin pump breaks), *reassurance of worth* (e.g., recognition of one’s competence found during times struggling with blood sugar readings that are out of range), *attachment* (e.g., emotional closeness with group members and group as a whole), *social integration* (e.g., a sense of belonging to a group of social media acquaintances), and *opportunity for nurturance* (e.g., providing assistance to others).

## Future Directions

Future research should investigate health information seeking across different social media platforms including Instagram for review of health information sharing endorsed by influencers who are sponsored by pharmaceutical companies. Additional research should examine information seeking by caregivers of adolescents with T1D as much of T1D management is shared with family.

## Limitations

Due to limitations of online qualitative data collection, some qualitative responses were very brief and some participants did not answer some prompts at all. Future studies should delineate between seeking advice *versus* providing advice as many participants stated that they did not seek advice but instead offered it. The sample was cross-sectional and used convenience sampling. The sample was recruited from the DOC, which introduces the possibility of sampling bias. As to be expected, the online sample was mostly white, well-educated and female. A potential bias may be sampling of more active *versus* less active users in the DOC. Importantly, these results should not be generalized to other types of diabetes because each type of diabetes differs in its treatment (19).

## Conclusions

In conclusion, this project’s findings provide support for the relationships between seeking health information online, social support and T1D related health outcomes and behaviors. This project adds to the information seeking knowledge base by characterizing how individuals with T1D are using social media. With a better understanding of the roles of online social support and seeking health information online on disease management, this project serves as the first of several series of studies to improve usage of the DOC and facilitate constructions of interventions that encourage or discourage specific aspects of each behavior. Future research should seek to collect additional data to bolster validity and reliability for the developing scale. Currently, the scale is being tested in varying groups of the DOC. Despite many established psychosocial benefits to participating in online support groups and also physical benefits to the information being brokered in the online community, this community (and precisely, particular subgroups) may not benefit everyone with T1D.

## Data Availability Statement

The raw data supporting the conclusions of this article will be made available by the authors, without undue reservation.

## Ethics Statement

The studies involving human participants were reviewed and approved by The University of Texas at El Paso - 1216875-2. The participants provided their written informed consent to participate in this study.

## Author Contributions

AH and OM conceived of the presented idea. AH developed the study and recruited participants. DG and IS verified the analytical methods. All authors contributed to the article and approved the submitted version.

## Conflict of Interest

The authors declare that the research was conducted in the absence of any commercial or financial relationships that could be construed as a potential conflict of interest.

## Publisher’s Note

All claims expressed in this article are solely those of the authors and do not necessarily represent those of their affiliated organizations, or those of the publisher, the editors and the reviewers. Any product that may be evaluated in this article, or claim that may be made by its manufacturer, is not guaranteed or endorsed by the publisher.
